# Treatment of Scarlatina

**Published:** 1812-02

**Authors:** 


*To the Editors of the Medical and Physical Journal.*

GENTLEMEN,

HAVING seen several letters in your Journal respecting the manner of treating Scarlatina, I beg leave to mention the following practice, which has been successfully pursued in a populous town in the south-east of Kent. In August and September the Scarlatina simplex and anginosa became very general; in the former we prescribed an emetic at the commencement, which, with due attention to the non-naturals, and a little aperient medicine (the Sub. Mur. Hydrarg. and Pulv. Jalapi), when on the decline, was all we found necessary; we treated S. anginosa in the same manner, with the addition of blistering, and leeches to the neck, scarifying the tonsils, inhaling the steam of warm vinegar and water, and gargling the throat with Potass. Oxymur. ʒj. Aq. Pure Acid. Muriatic. ā ℥j. M. ℞. Hujus liquoris gt. xxx. Mit. Camphora ℥iv. M. which we likewise gave as recommended by Mr. T. A. Braithwaite, in Duncan's Annals of Medicine for 1804. If the febrile symptoms ran high, and delirium attended, we gave the common saline mixture, and ordered warm pediluvium; when the fever was subdued, tonics were administered if necessary. By the above treatment, as the peculiar symptoms indicated, we did not lose a single patient.

I am, Gentlemen,

Your obedient Servant,

T. E. B.

In your Journal, No. 150, p. 115, is a letter by Mr. Fogo, containing some remarks, for which I am desirous of farther authority than his mere *ipse dixit*. He says, "I may farther presume to add, if he (the Essex Gentleman) had been of his present opinion, and left the second to nature, other two hours he would very probably have witnessed the death of both mother and child." Now I think it equally probable he would not; at the time of his delivering the second, the effusion of blood already taken place must have *nearly* filled the uterus, even supposing it to have not been partially contracted, and by pressing on the mouths of the vessels must have prevented any farther hæmorrhage, in the same manner as plugging the vagina in severe floodings. From his remarks, it is evident he is in the habit of delivering the second immediately *per artem*, which is not a practice usually recommended. Burns advises us to wait three or four hours, when, if no effective pains come on, to deliver; many eminent practitioners in London inculcate the same doctrine.

doctrine. I am acquainted with a gentleman in extensive practice, who never interferes except in preternatural presentations; and in two or three instances waited *twelve* hours without any ill consequence ensuing. Opposing the passage of the child is a measure I would strongly recommend, having known syncope, and irregular contraction of the uterus, take place from its too sudden expulsion. Guarding the perinæum is a practice I should not have imagined any person could have objected to; as laceration has sometimes, though certainly very rarely, taken place, there can be no harm in endeavoring to prevent it. I hope Mr. Fogo will favor us with some farther remarks on this interesting subject, as I must confess myself not hitherto a convert to his doctrine.

